# Primary Cutaneous Nocardiosis in a Patient Taking Adalimumab Therapy for Crohn’s Disease

**Published:** 2017-02-15

**Authors:** Madhav Desai, Muhammad Imran, Ayesha Irum, Julian Magadan

**Affiliations:** 1 Department of Internal Medicine, University of Kansas Medical Center, Kansas City, KS; 2 Division of Allergy, Clinical Immunology and Rheumatology, University of Kansas Medical Center, Kansas City, KS; 3 Army Medical College, Rawalpindi, Pakistan

**Keywords:** nocardia infections, adalimumab, Crohn’s disease

## Introduction

The introduction of biologic immunomodulators, in particular antibodies targeted against tumor necrosis factor alpha (TNF-alpha), has revolutionized treatment of Crohn’s disease. However, this comes at the expense of a higher risk for opportunistic infections due to a generalized immunosuppressive effect. Bacterial and opportunistic infections are well-known complications of anti-TNF agents.^1^,^2^ Nocardia has been reported rarely among patients on anti-TNF agents.^3^

TNF plays a role in the clearance of norcardia in animal models.^4^ Immunosuppression, anti-TNF treatment in particular, may favor growth and dissemination of nocardia. Pulmonary and cutaneous nocardiosis has been reported in Crohn’s disease patients on infliximab.^5^,^6^ Nocardiosis has been reported with adalimumab therapy in rheumatoid arthritis patients.^7^,^8^ Our search did not reveal any cases of nocardiosis while on adalimumab therapy in Crohn’s disease patients.

Crohn’s disease patients receiving biologic agents, in particular, tumor necrosis factor (TNF)-alpha inhibitors are immunosuppressed and are prone to develop opportunistic infections. We report a rare case of primary cutaneous nocardiosis in an immunocompromised patient on chronic anti-TNF for underlying severe Crohn’s disease.

## Case Report

**Figure 1:**
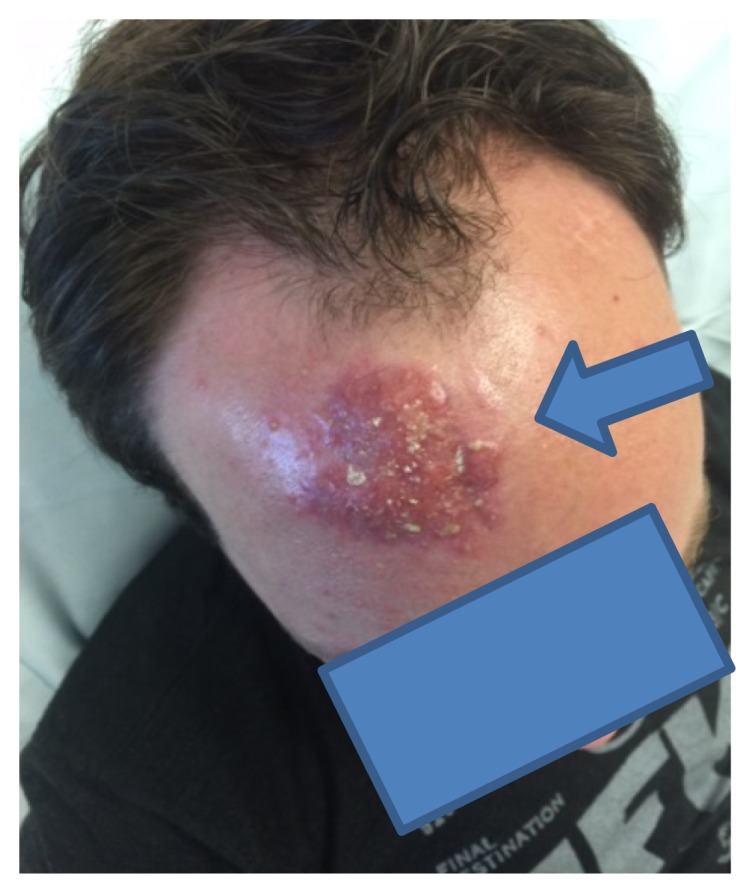
Cellulitis-like rash with draining and crusting.

A 36-year-old Caucasian gentleman with a history of Crohn’s disease treated with adalimumab presented with a cellulitis-like rash on his forehead (Figure 1). Five weeks previously, he had struck his forehead on a construction pole, sustaining a laceration which required sutures. On presentation he had developed purulent discharge from the laceration site, and he was started on doxycycline. After completing antibiotic treatment, he was seen by a dermatologist due to persistent rash and a punch biopsy was taken from the area.

Histological examination of the biopsy showed granulomatous inflammation thought to be related to a foreign body. The lesion worsened with increasing pain, pruritus, erythema, and development of a furuncle with drainage of yellow purulent material. A swab was sent for aerobic culture which grew coagulase negative staphylococci. Viral and fungal cultures from this lesion were negative.

**Figure 2:**
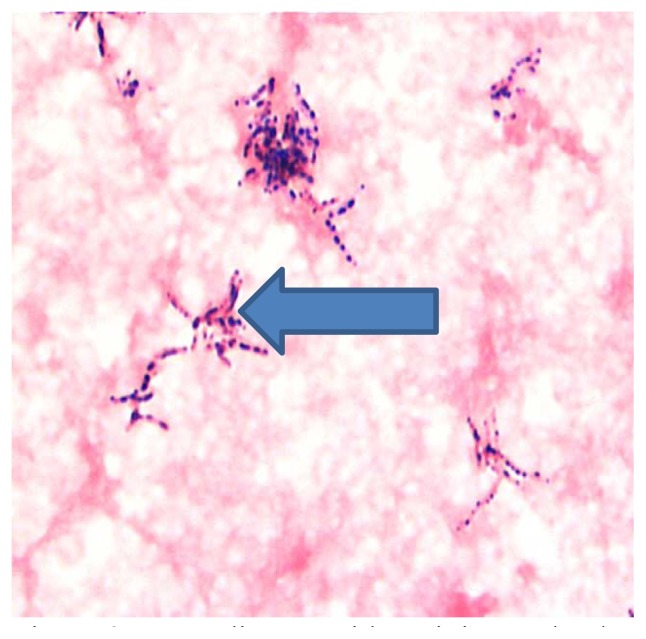
Nocardia asteroids staining and culture.

Over the next several weeks, the lesion continued to worsen, growing in size and becoming more painful. The adalimumab therapy was stopped and pyoderma gangrenosum was considered due to the patient’s underlying Crohn’s disease. The patient subsequently was admitted due to uncontrolled pain, and progression of his forehead lesion. A comprehensive work-up for human immunodeficiency virus, syphilis, tuberculosis, hepatitis, antinuclear antibody, rheumatoid factor, anti-cyclic citrullinated peptide, and serum protein electrophoresis were negative. Blood (bacterial and fungal), urine, and sputum cultures were negative. Chest x-ray was negative. With IV antibiotics, there was some improvement in the forehead rash and the patient was discharged on oral antibiotics. He also was started on dapsone and clobetasol for treatment of pyoderma gangrenosum. One week later, cultures from his biopsy grew *Nocardia arthritidis* (Figure 2). He was started on trimethoprim-sulfamethoxazole daily with follow-up with an infectious disease specialist. His rash improved significantly after a few weeks of treatment.

## Discussion

Human nocardiosis is caused by nocardia species which is an ubiquitous soil inhabiting bacteria and considered an opportunistic pathogen.^8^ It can affect the skin and classically disseminate to involve the lungs and brain. It is a difficult infection to treat and carries high mortality if disseminated.^9^ Nocardiosis has been well known to affect AIDS patients, transplant recipients, and long term corticosteroid treated individuals. Now, it is being recognized increasingly in immunosuppressed patients on anti-TNF agents. Previous use of corticosteroids has been identified as a risk factor, as noted in our case, and was present in more than 50% of cases in earlier studies.^10^

Early diagnosis is essential and norcardia should be considered in the differential diagnosis of any patient presenting with draining skin lesion or painful rash who is receiving anti-TNF agents or has in the recent past. Anti-TNF therapy doubles the risk of opportunistic infections in inflammatory bowel disease patients.^9^,^10^ This underscores the importance of adherence to guidelines for their prevention and management. A high level of vigilance and scrutiny in examination and follow up are highly recommended for this group of patients.

## Conclusion

A high level of suspicion must be enforced when patients receiving TNF-alpha inhibitors present with skin manifestations, in particular, cellulitis-rash or draining lesion. Early identification and treatment of nocardia is pivotal to prevent disseminated disease and mortality.
